# Yoga as an Adjunct for Management of Opioid Dependence Syndrome: A Nine-Month Follow-Up Case Report

**DOI:** 10.1155/2021/5541995

**Published:** 2021-07-23

**Authors:** Prateek Varshney, Hemant Bhargav, Pilli Devi Vidyasagar, Sumana Venugopal, Rashmi Arsappa, Venkata Lakshmi Narasimha, Priyamvada Sharma, Vijayashree Rao, Pratima Murthy

**Affiliations:** ^1^Department of Psychiatry, National Institute of Mental Health and Neurosciences (NIMHANS), Hosur Main Road, Bengaluru 560029, India; ^2^Department of Integrative Medicine, National Institute of Mental Health and Neurosciences (NIMHANS), Hosur Main Road, Bengaluru 560029, India; ^3^Centre for Addiction Medicine, Department of Psychiatry, National Institute of Mental Health and Neurosciences (NIMHANS), Hosur Main Road, Bengaluru 560029, India; ^4^Department of Clinical Psychopharmacology and Neurotoxicology, NIMHANS, Bengaluru, India

## Abstract

Opioid dependence syndrome (ODS) is a chronic relapsing remitting condition associated with significant impairment and mortality risk. Opioid substitution therapy is used worldwide, but long-term retention rates are low and there is risk of misuse and diversion. Yoga practice can improve quality of life, reduce chronic pain, and enhance endogenous opioids (beta-endorphins). We describe a case of ODS where yoga was added to the conventional management and who was followed up for 9 months. Assessments were done for clinical symptoms, urine drug screening, plasma beta-endorphins, and Buprenorphine dosage. We observed an improvement in his clinical symptoms and reduction in the requirements for Buprenorphine. A slight increase in basal plasma beta-endorphin levels was also observed at the 9-month follow-up (from 2.02 pmol/L at baseline to 6.51 pmol/L).

## 1. Introduction

Opioid dependence syndrome (ODS) is a major public health concern as it results in enhanced risk of death due to overdose, deterioration in quality of life, and economic burden to the society [1].

Buprenorphine (BPN) maintenance treatment and “opioid substitution” are recommended as the first line of management for opioid dependence [2]. While “opioid substitution therapy (OST)” is an evidence-based harm reduction strategy, it poses significant challenges due to associated financial burden, side effects, risk of misuse and diversion, and low long-term retention rates [3]. Brain-related changes of addiction continue to exist despite protracted BPN maintenance treatment [4].

Endogenous opioids are present in the body, which play a role in the substance addictive process [5]. Several studies suggest that yoga, as a comprehensive mind-body program, may regulate pain behaviors [6] and modulate the endogenous opioid system through the release of endorphins (R. K. [7]). Thus, yoga may be a useful cost-effective and indigenous adjunct intervention that can add much-needed component of holistic lifestyle management in ODS [8].

## 2. Case Report

A 33-year-old married male, from urban Manipur, of middle socioeconomic status, diploma holder in pharmacology and unemployed (previously working as a pharmacist), was admitted in our tertiary psychiatric facility. He presented with insidious onset and continuous progressive illness of 8-year duration, characterized by heroin use in a dependence pattern, associated with harmful use of tobacco (cigarette smoking). The first exposure to heroin was 8 years back as an experiment with friends. After a month of daily use of injectable heroin, the patient started buying heroin for himself spending up to Rs 500 per day (which was 90% of his average daily income). He started using heroin alone, up to 0.5 gram per day, which was more than twice the amount of initial use, to get the desired effects of relaxation and euphoria. Over the next 2 years, he started to develop withdrawal symptoms after 6-9 hours if he did not take heroin. These symptoms included body ache, back pain, runny nose, stomach cramps, feeling restless, with lack of appetite and sleep, and constipation. The patient had been married during this time but was unable to abstain from heroin consumption despite his spouse's multiple pleas. He was therefore admitted to a deaddiction facility where he stayed for 1.5 months. However, he relapsed within a week postdischarge due to craving. In the last 8 years, there were four episodes of overdose requiring hospitalization, and several of his friends expired due to heroin overdose during this time. However, he was unable to refrain from the same, with a maximum period of abstinence being 1.5 months and three previous relapses. Thus, the patient was admitted at our center and started on opioid substitution therapy after obtaining informed consent. At the time of admission, multiple injection marks with thrombosed veins were noted on his upper limbs, and he was in a preparation stage of motivation.

The patient was started on 2 mg BPN built over 1 week to 18 mg along with nicotine patch 21 mg daily. From the third day of his admission, the patient was referred to the yoga facility in the institute. A 60-minute validated yoga program for ODS was included as an adjunct in his routine [9]. The program included practices of physical postures, sun salutations, relaxation techniques, regulated fast and slow yogic breathing practices, mantra chants, and yoga-based lifestyle counselling. One to one supervised yoga was taught for 5 days per week for the first 2 weeks, followed by tele-yoga practice of the same module (3 times per week) plus home practice postdischarge (with recorded videos) up to the 3^rd^ month. After that, he continued practicing yoga on his own. An online booster session was offered to the patient every month after the 3^rd^ month. The patient was followed up on a monthly basis over phone and was motivated to continue practicing yoga.

To evaluate how well the patient learnt yoga, the yoga performance assessment scale (YPA) [10] was applied by the yoga therapist on each follow-up assessment. Plasma beta-endorphin levels were also assessed at baseline, after 2 weeks, and at the end of 9 months. The patient was followed up for the next 9 months with assessments of several clinical parameters including Buprenorphine dosage. Plasma beta-endorphin levels were also assessed at the baseline and after 2 weeks of supervised practice. Clinical assessments were performed by a psychiatrist. Standard procedures were followed for assessing endorphin levels [11]. Written informed consent was obtained from the patient.

## 3. Results

The patient was discharged after 2 weeks of supervised yoga. During this time, his BPN dosage was increased from 18 mg to 24 mg (through fixed clinical protocol irrespective of his symptom status). His basal plasma endorphin levels increased from 2.02 pmol/L to 2.15 pmol/L in these 2 weeks. At the time of discharge, the patient was asked to continue practicing the 45-minute yoga module (using a recorded video) for at least 5 days per week. Three supervised tele-yoga sessions were conducted per week up to 3 months. At the end of 3 months, he showed 65% adherence to the yoga program (i.e., practicing 65% of the recommended number of yoga sessions as per the daily attendance record that was signed by the patient and countersigned by the caregiver after the yoga practice) and his BPN dosage reduced further from 24 mg (at the time of hospital discharge) to 6 mg. In the next 2 months, he gradually stopped BPN on his own due to perceived risk of addiction to the same but continued to practice yoga. However, the patient could not reduce his smoking habit and continued to smoke 6-8 cigarettes/day. He was offered nicotine replacement therapy at the time of discharge but he decided not to continue the same. At the 3^rd^ month and 9^th^ month follow-up, his urine screening was negative for opioids. We also observed sustained improvements in his mood, anxiety, pain, sleep, appetite, sexual functions, and craving at the 3^rd^ month and 9^th^ month follow-ups (Table 1). Plasma endorphin levels could not be assessed on the next follow-up as it was difficult to draw blood from damaged veins of the subject each time, and the subject did not consent for the same. At the 9^th^ month, the basal plasma endorphin levels further increased to 6.51 pmol/L. The YPA scale showed that the patient was able to learn the module correctly after 10 supervised sessions of yoga in the first 2 weeks (93.3% score at the end of 2 weeks as compared to 50% score after the first session, Table 1). This performance was maintained on subsequent follow-ups at the 3^rd^ month, 6^th^ month, and 9^th^ month.

Since the last 4 months, the patient has not required any BPN and he is managing only with yogic practices. The patient reported that especially fast breathing practice of “Bhastrika” (bellows breath 20 strokes/cycle—3 cycles per day), regulated breathing (*vibhagiya pranayama*), and mantra chanting (sounds aa, uu, and mmm with sound resonance) helped him in (1) relaxing better, (2) improving concentration, and (3) controlling impulsivity. He does not report of craving or withdrawal symptoms at present and has regained adequate biological rhythm and social and occupational functioning with euthymic mood.

## 4. Discussion

We observed that the yoga program was a useful adjunct to BPN in managing this case. Previously, in an RCT on 55 male opioid-dependent subjects, Dhawan et al. observed that the adjuvant Sudarshan Kriya yoga program led to improved quality of life at the end of 6 months as compared to the as-usual treatment arm [12]. Another RCT of yoga on 75 female patients undergoing heroin detoxification in China revealed that 6 months of yoga practice improved mood states and quality of life better than the controls [13]. Current study also reports similar findings. In addition, we observed a small increase in plasma beta-endorphin levels after 9 months of yoga practice in this patient with ODS. Though such results with yoga intervention of 10 days have been observed before in patients with other noncommunicable diseases (A. [14]), present study study is the first attempt to report the same in ODS. In addition, we provide clinical data on long-term follow-up of 9 months for this patient.

Based on clinical practice and literature, the combination of opioid substitution therapies and psychotherapy are faced with challenges of low long-term retention rates in ODS [3]. Interdisciplinary rehabilitation programs that integrate yoga-based lifestyle may have the potential to provide long-term changes in improved physical and psychological well-being and personal empowerment. Future studies should explore this hypothesis using a robust design, with active controls and long-term follow-ups.

## Figures and Tables

**Table 1:** Changes in clinical and biological variables at baseline and on subsequent follow-up with add-on yoga therapy to conventional care.

S. no.	Variable	Baseline	15 days	3 months	9 months
1	Buprenorphine dosage (mg)	18	24	6	0
2	HAM A	21	6	4	2
3	HAM D	20	4	3	0
4	COWS	16	4	2	3
5	Fagerstrom test	8	NA	4	6
6	Sexual dysfunction (SDQ)	69	NA	51	34
7	Sleep latency (min)	60	10	10	30
8	Hours of sleep (hrs)	5	8	8	8
9	Pain VAS	8	3	3	0
10	Craving VAS	10	2	0	0
11	Constipation VAS	5	2	0	0
12	Restlessness VAS	10	5	1	0
13	Appetite VAS	4	6	6	10
14	CGI-S	4	2	2	1
15	CGI-I	0	2	2	1
16	YPA	16	28	30	28
17	Urine screening for opioids	Positive	NA	Negative	Negative
18	Plasma beta-endorphin levels (pmol/L)	2.02	2.15	NA	6.51

HAM A: Hamilton Anxiety Rating Scale; HAM D: Hamilton Depression Rating Scale; COWS: Clinical Opioid Withdrawal Scale; SDQ: Sexual Dysfunction Questionnaire; VAS: visual analogue scale; CGI-S: Clinical Global Impressions-Severity of Illness; CGI-I: Clinical Global Impressions-Global Improvement; YPA: yoga performance assessment scale; NA: not applicable/not available.

## Data Availability

Data can be shared by the corresponding author on request.
